# Genome-wide analysis of sulfur-encoding biosynthetic genes in rice (*Oryza sativa* L.) with *Arabidopsis* as the sulfur-dependent model plant

**DOI:** 10.1038/s41598-022-18068-0

**Published:** 2022-08-15

**Authors:** Muhammad-Redha Abdullah-Zawawi, Nisha Govender, Nor Azlan Nor Muhammad, Norfarhan Mohd-Assaad, Zamri Zainal, Zeti-Azura Mohamed-Hussein

**Affiliations:** 1 Institute of Systems Biology (INBIOSIS), Universiti Kebangsaan Malaysia, 43600 Bangi, Selangor Malaysia; 2 UKM Medical Molecular Biology Institute (UMBI), UKM Medical Centre, Jalan Yaíacob Latiff, Bandar Tun Razak, 56000 Cheras, Kuala Lumpur Malaysia; 3 Faculty of Science and Technology, Universiti Kebangsaan Malaysia, 43600 Bangi, Selangor Malaysia

**Keywords:** Computational biology and bioinformatics, Plant sciences

## Abstract

Sulfur is an essential element required for plant growth and development, physiological processes and stress responses. Sulfur-encoding biosynthetic genes are involved in the primary sulfur assimilation pathway, regulating various mechanisms at the gene, cellular and system levels, and in the biosynthesis of sulfur-containing compounds (SCCs). In this study, the SCC-encoding biosynthetic genes in rice were identified using a sulfur-dependent model plant, the *Arabidopsis*. A total of 139 *At*SCC from *Arabidopsis* were used as reference sequences in search of putative rice SCCs. At similarity index > 30%, the similarity search against *Arabidopsis* SCC query sequences identified 665 putative *Os*SCC genes in rice. The gene synteny analysis showed a total of 477 syntenic gene pairs comprised of 89 *At*SCC and 265 *Os*SCC biosynthetic genes in *Arabidopsis* and rice, respectively. Phylogenetic tree of the collated (*At*SCCs and *Os*SCCs) SCC-encoding biosynthetic genes were divided into 11 different clades of various sizes comprised of branches of subclades. In clade 1, nearing equal representation of *Os*SCC and *At*SCC biosynthetic genes imply the most ancestral lineage. A total of 25 candidate *Arabidopsis* SCC homologs were identified in rice. The gene ontology enrichment analysis showed that the rice-*Arabidopsis* SCC homologs were significantly enriched in the following terms at false discovery rate (FDR) < 0.05: (i) biological process; sulfur compound metabolic process and organic acid metabolic processes, (ii) molecular function; oxidoreductase activity, acting on paired donors with incorporation or reduction of molecular oxygen and (iii) KEGG pathway; metabolic pathways and biosynthesis of secondary metabolites. At less than five duplicated blocks of separation, no tandem duplications were observed among the SCC biosynthetic genes distributed in rice chromosomes. The comprehensive rice SCC gene description entailing syntenic events with Arabidopsis, motif distribution and chromosomal mapping of the present findings offer a foundation for rice SCC gene functional studies and advanced strategic rice breeding.

## Introduction

Sulfur (S) is an important macronutrient for plant growth and development, immunity, and stress mitigation. In sulfur-deficient soils, plants invoke stress resistance and xenobiotic detoxification^1,2^. Plant S assimilation is translated into sulfur-containing compounds (SCCs), a class of important secondary metabolites. Plants utilize freely available sulfate in the soil to synthesize SCCs for growth and functional metabolisms. The primary S assimilation pathway integrates carbon, nitrogen, and S for the synthesis of various SCCs such as glutathione, S-adenosylmethionine, S-methylmethionine, sulfoquinovosyldiacylglycerol, ferredoxin and thiol-group containing plant defensins^3^. Both the thioredoxins and gluthathiones are redox modulators with detoxifying abilities. In view of the ecological perspective, various vital biological functions which include oxidative stress mitigation, heavy-metal detoxification^4,5^ and plant defense responses against biotic factors^2,6^ are regulated by SCCs.

Rice (*Oryza sativa* L.) is the second most preferred food crop consumed worldwide, after wheat. Cultivated in over 114 countries around the world, rice feeds half the world population (3 billion people) and warrants global food security^7^. It is predicted that rice production exceeding 800 million tonnes is required to meet the calorie demand of the expected world population in 2025^8^. With climate change in the chart of global issues, abiotic stresses are strongly impacting rice productivity. Major limiting factors in the rice production system includes drought, heat, cold and salinity. In others, waterlogged paddy soils inherent toxic elements such as Cd, As and Fe. Rapid response to stressors regulates stress mitigation responses which include transmembrane transport, glutathione metabolism, signal transduction, and redox control^9^. In rice, S-associated genes, metabolites and proteins have shown involvement in abiotic stress responses and mitigation. For example, in Cd and As co-contaminated soils, the glutathione metabolism-related genes (*Oso1g05367700* and *Oso1g0530900*) were significantly up-regulated relative to the control conditions. During rice drought stress response, the glutathione S-transferase activities were significant increased^10^. In another study, glutathione peroxidases and thiol-based antioxidant enzymes regulated the ABA-independent osmotic stress signalling in rice^11^. Although the role of SCC-encoding genes and SCCs in rice stress response have been documented by numerous studies, little is known about the SCC gene distribution and pattern, and putative functions at the rice genome scale. The SCC genome-level information is important to shed new information and knowledge in innovative rice breeding strategies.

Plant SCC distribution varies greatly with species. In the Brassicaceae family, more than two hundred different types of glucosinolates (GLSs) with potent roles in defense responses have been reported^12,13^. The GLS-myrosinase defense system gets activated during a pathogen attack to form unstable aglycone intermediates. Thereafter, a range of toxic volatile compounds (isothiocyanates, nitriles, and thiocyanates) is produced during hydrolysis for deterrence against the invading pathogen/pests^14^. In others, camalexin, an indole-type phytoalexin SCC is produced for adaptivity against abiotic stress and pathogen attack, alike^2^. Camalexin derived from tryptophan is converted to indole-3-acetaldoxime, which later switches into indole-3-acetonitrile upon dehydration^15^. *Arabidopsis* (Brassicaceae) and rice from the grass family (Poaceae) are S-dependent families. With about 10–30% of S expressed in the plant tissues, the first is ranked as the most S-dependent family^3,16–19^.

In this study, the SCC-encoding biosynthetic genes in rice are identified and characterized using *Arabidopsis* as the reference genome model of an S-dependent plant family. The Arabidopsis genome is an excellent reference for the identification of S-encoding biosynthetic genes in rice. There is a burst of SCC-related functional experiments and databases^20,21^ extensively reported in *Arabidopsis*; low-affinity sulphate transporters^22^; S dioxygenase activity in ETHE1 knockout mutant^23^; S deficiency responsive genes^24^; *Arabidopsis* S metabolome^25^; S-containing secondary metabolites from *Arabidopsis*^2^. The synteny and similarity of the *Arabidopsis*-rice SCC homologous sequences are visualized and the enrichment analysis along a cross-comparison of the corresponding motif sequences is provided to gain information on the extent of similarities. The findings extent to compare and capture the *Arabidopsis*-rice evolutionary relationship, predict the ecological functions of SCC genes in rice and provide the genetic basis for stress mitigation and defense response enhancement in rice breeding.

## Materials and methods

### *Arabidopsis* and rice genome sequences

*Arabidopsis thaliana* and *O. sativa* genome sequences and genome annotations were obtained from the Phytozome v13.0 database (https://phytozome-next.jgi.doe.gov/)^26^, *Arabidopsis* Information Resource (TAIR) v10.0 (https://www.arabidopsis.org)^27^ and *O. sativa* Genome Annotation Project Database (RGAP) v7.0 (http://rice.uga.edu/)^28^. The *Arabidopsis* genome was set as reference sequence against the rice (query) sequences.

### Sulfur-containing compound (SCC)-encoding biosynthetic gene mining

The SCC-encoding genes in *Arabidopsis* (*At*SCC) were mined from AraCyc version 14.0 (https://pmn.plantcyc.org/)^29^ using the following keywords: (i) glucosinolate, and (ii) camalexin. The *At*SCC biosynthetic protein sequences were designated as query for the identification of corresponding homologs (*O. sativa* SCC biosynthetic genes) in the rice genome using the BLAST program (http://blast.ncbi.nlm.nih.gov)^30^. Reciprocal searching was applied using BLASTP default parameters: e-value = 1e-10 and sequence similarity > 30%. The gene positions were determined by parsing the genome annotation file and the BLAST output. The genomic feature information (General Feature Format) file was concatenated as the input data for subsequent analysis.

### Synteny analysis

The Multiple Collinearity Scan Toolkit X software (MCScanX) was employed for the identification of collinear blocks of homologous sequences and multiple alignment of collinear blocks to the chromosomes. Input files were executed by the MCScan function and the expected number of occurrences (E) of the collinear blocks was calculated^31^. The following default parameters were applied: E-value cut-off = 1e-05 and match_size = 5. The collinear blocks of interspecies were labelled as *AtSCC* and *OsSCC*, denoting *A. thaliana* and *O. sativa*, respectively. All rice-*Arabidopsis* collinear blocks of gene pairs (two interspecies chromosomal positions) were identified and visualized using Rcircos software^32^.

### Multiple sequence alignment and phylogenetic analysis

A multiple sequence alignment of the rice-*Arabidopsis* SCC-encoding biosynthetic genes was performed using the Multiple Sequence Comparison by Log-Expectation (MUSCLE) (https://www.ebi.ac.uk/Tools/msa/muscle/) with the following settings: gap open penalty =  − 2.9, gap extension = 0, and hydrophobicity multiplier = 1.2^33^. Phylogenetic analysis was performed using Molecular Evolutionary Genetics Analysis (MEGA) v7.0 (http://megasoftware.net)^34^. The maximum-likelihood (ML) by Tamura-Nei substitution model and phylogeny test using 1000 replicates of the bootstrap method were applied. The ML phylogenetic tree was visualized and annotated using the Interactive Tree Of Life (iTOL) v4.0 (http://itol.embl.de)^35^.

### Motifs search distributions, gene structure analysis and chromosomal mapping

The exon–intron architecture of *At*SCC and *Os*SCC biosynthetic genes was visualized using the Gene Structure Display Server 2.0^36^. Conserved motifs were identified using the Multiple Expectation Maximization for Motif Elicitation (MEME) v4.11.3 (http://meme-suite.org/) tool with the following parameters: the number of motifs = 10, motif site distributions mode = 0/1 occurrence per sequence (zoops)^37^. The consensus motif sequences were annotated using Database of protein domains, families and functional sites (PROSITE) (https://prosite.expasy.org)^38^, Pfam, database for protein families v35.0 (http://pfam.xfam.org/)^39^ and Conserved Domain Database v3.19 (CDD) (https://www.ncbi.nlm.nih.gov/Structure/cdd/cdd.shtml))^40^. The chromosoma gene loci were mapped using the Chromosome Map Tools available in TAIR (https://www.arabidopsis.org/jsp/ChromosomeMap/tool.jsp)^41^ and Oryzabase (http://viewer.shigen.info/oryzavw/maptool/MapTool.do)^42^ of *A. thaliana* and *O. sativa* genes, respectively. Genes separated by less than five genetic loci within 5 to 100 kb were scored as tandem duplications.

### Gene ontology (GO) enrichment and pathway

Functional enrichment analysis of the SCC-encoding biosynthetic genes (*At*SCC and *Os*SCC) was performed using ShinyGO v.0.75 (http://bioinformatics.sdstate.edu/go75/) with p-value cut-off set at false discovery rate (FDR) = 0.05: (i) Gene ontology classification^43^ and (ii) KEGG pathway enrichment^44^. The *A. thaliana* and *O. sativa* Japonica genomes were set as reference datasets. The 20 top-most significantly enriched *At*SCC and *Os*SCC genes were identified using the Venn webserver (https://bioinformatics.psb.ugent.be/webtools/Venn/).

## Results

### Identification of putative OsSCC biosynthetic genes using synteny analysis

**Figure 1:**
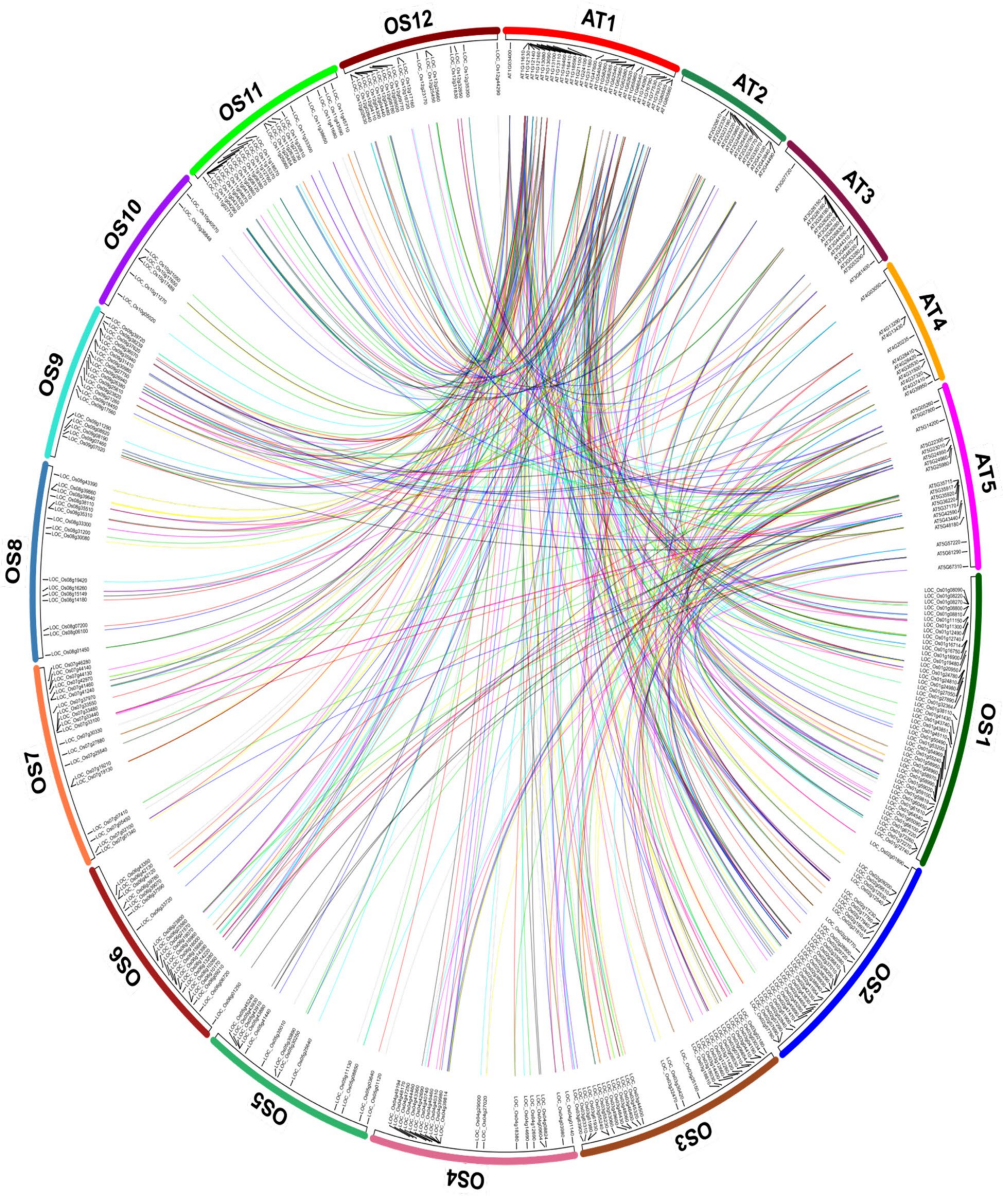
The *Arabidopsis thaliana* (At)-*Oryza sativa* (Os) sulfur-containing compound (SCC) encoding biosynthetic synteny gene pairs identified with MCScanX. There are 477 syntenic gene pairs (represented by connecting colour lines) between 89 AtSCC and 265 OsSCC biosynthetic genes. The numbering on AT and OS labels denotes the chromosome number.

A total of 139 *At*SCC biosynthetic genes were obtained from a rapid search performed with the following descriptions: (i) glucosinolate activation (herbivore attack and intact plant cell) pathways, (ii) aliphatic glucosinolate (derived from homomethionine, dihomomethionine, trihomomethionine, hexahomomethionine, pentahomomethionine, and tetrahomomethionine), (iii) indolic glucosinolate (tryptophan derivative), (iv) aromatic glucosinolate (phenylalanine derivative) and (v) camalexin. The sequence homology search identified a total of 838 SCC biosynthetic genes in *O. sativa*. A total of 173 sequences were discarded due to low sequence similarity (< 30%) and the remaining 665 candidates were subjected to synteny analysis. Under various combinations, a total of 477 syntenic gene pairs with 89 *At*SCC and 265 *Os*SCC biosynthetic genes were identified (Supplementary 1). The syntenic gene pairs were randomly distributed across the chromosomes with sizes, as annotated by the gene number (GN). In rice, the syntenic GN distribution were as following: *Os*Chr1; syntenic GN = 45, *Os*Chr2; syntenic GN = 32, *Os*Chr3; syntenic GN = 28, *Os*Chr11; syntenic GN = 23, *Os*Chr4, *Os*Chr 7 and *Os*Chr 9; syntenic GN = 20, *Os*Chr12; syntenic GN = 18, *Os*Chr8; syntenic GN = 16, and *Os*Chr5; GN = 13. In *A. thaliana*, the highest number of syntenic genes were distributed in *At*Chr1 (syntenic GN = 31), followed by *At*Chr5 (syntenic GN = 19), *At*Chr3 (syntenic GN = 15), *At*Chr2 (syntenic GN = 13) and *At*Chr4 (syntenic GN = 11) (Fig. 1).

The distribution of syntenic gene pairs (SPs) was higher in *At*Chr1 (SPs = 267) and *At*Chr5 (SPs = 113) in comparison to *At*Chr2 (SP = 37) and *At*Chr3 (SP = 33). Overall, a total of 41 *At*SCC and 25 *Os*SCC biosynthetic genes were linked with at least four synteny blocks. Ten *Os*SCC biosynthetic genes from the cytochrome P450 gene family with at least five or more synteny blocks were identified as following: CYP89D1 (*LOC_Os01g24810*), CYP706C2 (*LOC_Os01g50490*), CYP73A35P (*LOC_Os01g60450*), CYP71AA3 (*LOC_Os01g72740*), CYP71U3 (*LOC_Os02g17760*), CYP51H4 (*LOC_Os02g21810*), CYP73A40 (*LOC_Os02g26770*), CYP86E1 (*LOC_Os02g38290*), CYP81A6 (*LOC_Os03g55240*) and CYP735A4 (*LOC_Os09g23820*) (Supplementary 1).

### Phylogenetic analysis of the SCC biosynthetic genes in *A. thaliana* and *O. sativa*

**Figure 2:**
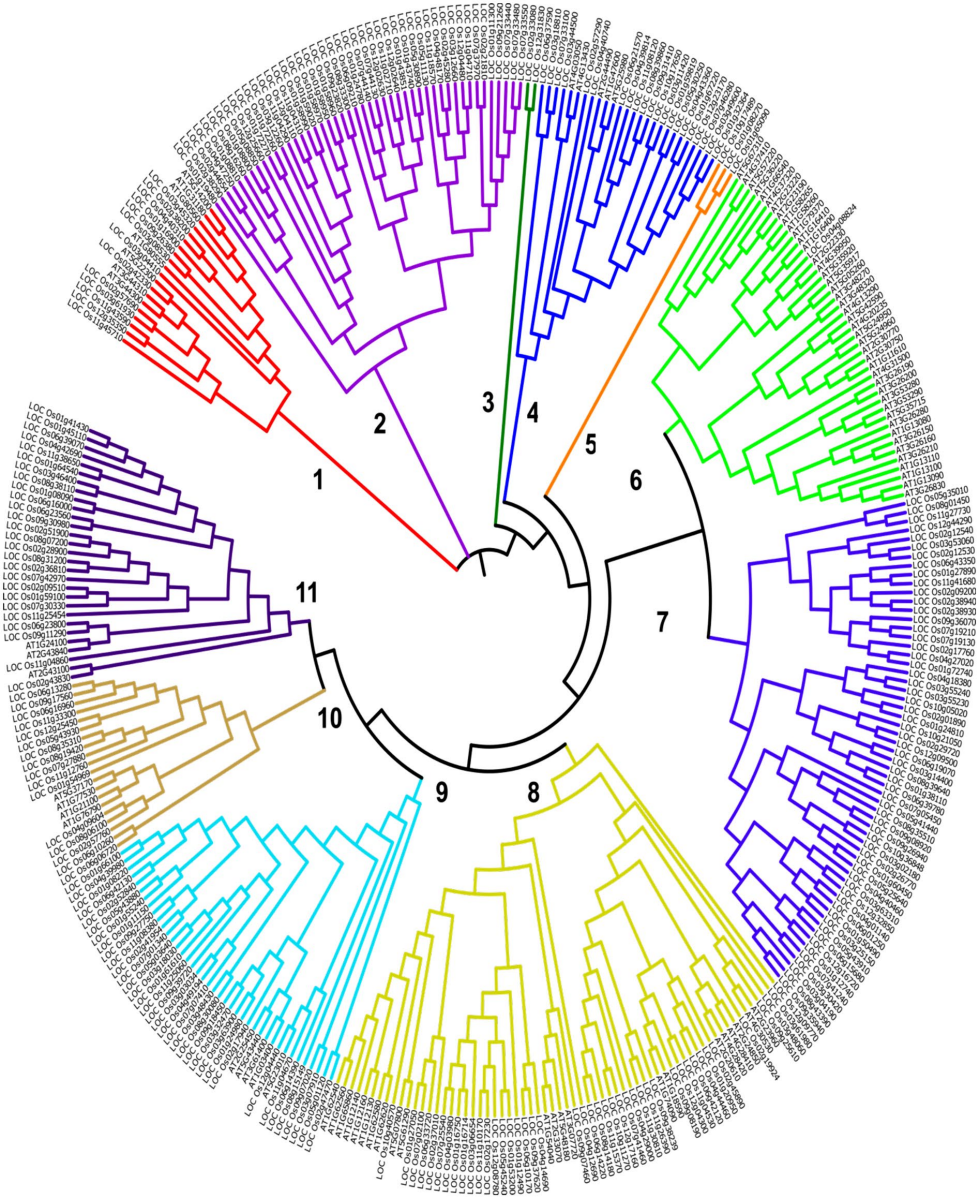
Phylogenetic analysis of collated sulfur-encoding biosynthetic genes in *Arabidopsis thaliana* and rice (*Oryza sativa*). The tree is constructed with MEGA software.

The phylogenetic tree comprised of 89 *At*SCC and 265 *Os*SCC biosynthetic genes show 11 different clades of various sizes, as annotated by the gene number (GN). Clade 8 emerged as the largest group with GN = 65, followed by clade 7 (GN = 59), clade 2 (GN = 46), clade 6 (GN = 44), clade 9 (GN = 40), clade 11 (GN = 29), clade 4 (GN = 26), and clade 1 and clade 10 with GN = 20, each. Clade 5 and clade 2 were the smallest in size, with GN = 3 and GN = 2, respectively. There were 7 clades comprised of *Os*SCC and *At*SCC biosynthetic genes in combination: clade 1, clade 4, clade 6, clade 8, clade 9, clade 10 and clade 11. Clade 1 showed nearing an equal number of *Os*SCC and *At*SCC biosynthetic genes. In clade 1, *At*NIT2 (*At3g44300*), *At*NIT1 (*At3g44310*), *At*NIT4 (*At5g22300*) and *Os*NRT2 (*LOC_Os02g42330*) were present together (Fig. 2).

In clade 4, synteny events between the *At*BGLU34 (*At1g47600*) biosynthetic gene and *Os*6BGLU24 (*LOC_Os06g21570*), *Os*4BGLU9 (*LOC_Os04g39814*), *Os*11BGLU37 (*LOC_Os11g08120*), *Os*8BGLU27 (*LOC_Os08g39860*) and *Os*9BGLU29 (*LOC_Os09g31410*) biosynthetic genes were identified. Likewise, clade 8 showed a collinear relationship between the *Os*SOT (*LOC_Os09g08190*) biosynthetic gene and the *At*SOT18 (*At1g74090*) biosynthetic gene. In clade 9, *At*ACO9 (*At5g43440*) was grouped together with *Os*2ODD25 (*LOC_Os03g32470*), *Os*FLS1 (*LOC_Os09g18450*), *Os*2ODD16 (*LOC_Os01g24980*), and *Os*2ODD26 (*LOC_Os03g63900*), whilst *Os*HIS1 (*LOC_Os02g17940*) was paired with *At*ACO4 (At1g03400) and *At*ACO8 (At3g61400). There were three syntenic pairs identified in clade 10: (i) *Os*COMTL4 (*LOC_Os02g57760*)-AtIGMT5 (*At1g76790*) biosynthetic genes, (ii) *Os*COMTL5 (*LOC_Os04g09604*)-AtIGMT1 (*At1g21100*) biosynthetic genes and, (iii) *Os*COMT (*LOC_Os08g06100*)-AtIGMT1 (*At1g21100*) biosynthetic genes. In clade 11, both *Os*GTF (*LOC Os11g04860*) and OsIAGLU (*LOC Os09g11290*) biosynthetic genes were identified as syntenic pairs of *At*UGT74B1 (*At1g24100*). No syntenic evidence was present in clade 6 (Figs. 1 and 2).

### Conserved motif analysis

**Figure 3:**
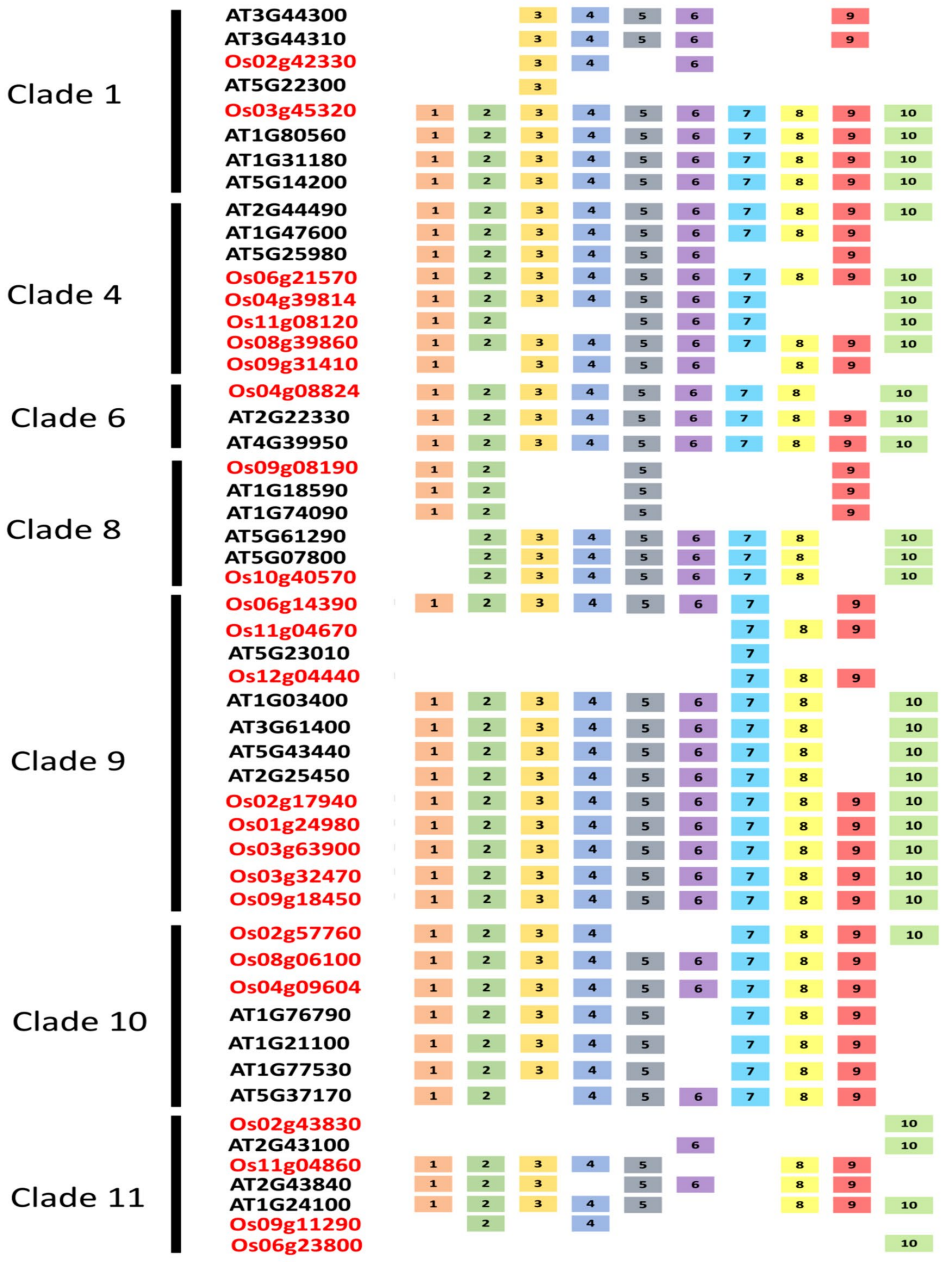
Motif distribution structure of *Arabidopsis thaliana* and *Oryza sativa* sulfur-encoding biosynthetic genes grouped by clades. The *A. thaliana* (ATXXXXXXX) and *O. sativa* (OsXXXXXXXX) gene IDs are written in black and red, respectively. Detailed information on the motif sequence information and annotation is available in Supplementary 2.

A total of ten conserved motifs were identified from *A. thaliana* and *O. sativa* SCC biosynthetic genes in clade 1, clade 4, clade 6, clade 8, clade 9, clade 10 and clade 11. The detailed motif sequence information and annotations are provided in Supplementary 2. The motif distribution was similar within the clade level. All the SCC-encoding biosynthetic genes contained at least one motif, whereas a total of 14 genes displayed all 10 motifs with mosaic patterning. No apparent pattern was observed among the motifs within the different species. The following motifs were annotated as isopropyl malate dehydrogenase (IPMDH): motifs 2, 3, 4, 7, 9 and 10. All the SCC-encoding biosynthetic genes in clade 4 displayed motif 6 (annotated as glucosidase) (Supplementary 2). Motif 1 and motif 5 contain the conserved sulfotransferase domain (Fig. 3). In clade 9, at least nine different motifs were consistently present in the member genes. Motifs 1, 2, 4 and 5 are annotated with the O-methyltransferase domain. The conserved motifs 1, 2, 4, 8 and 9 were also described as UDP-glycosyltransferase (Fig. 3) (Supplementary 2).

### The exon–intron structure of the SCC biosynthetic genes in *A. thaliana* and *O. sativa*

**Figure 4:**
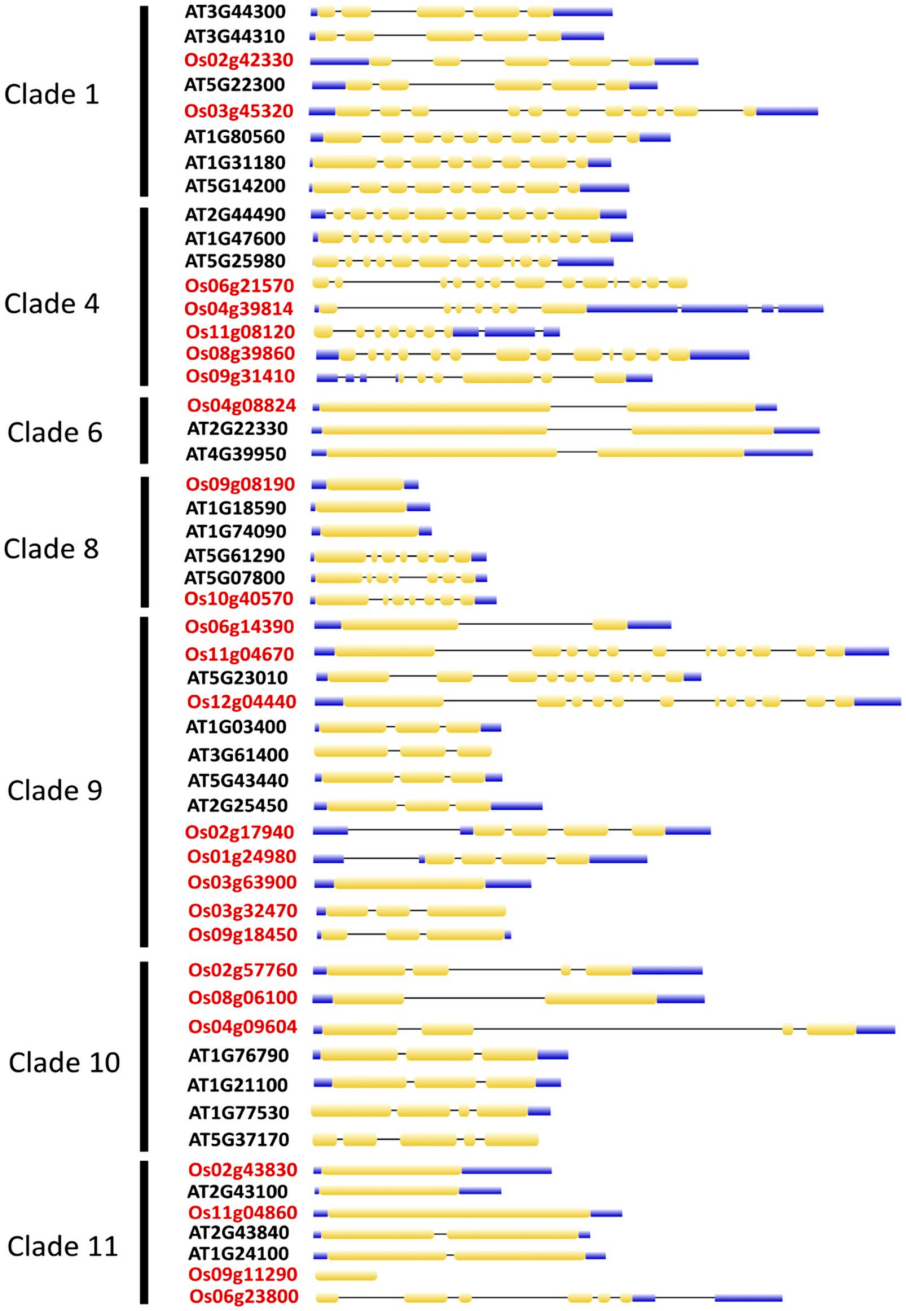
Illustration of the *Arabidopsis thaliana* and *Oryza sativa* sulfur-encoding biosynthetic gene structure. Genes are grouped according to clades. The *A. thaliana* (ATXXXXXXX) and *O. sativa* (OsXXXXXXXX) gene IDs are written in black and red, respectively. Exons are indicated as yellow round-corner rectangles and introns with solid black lines.

Generally, the number of exons (EN) and introns (IN) in *Arabidopsis* and rice displayed no apparent trend by species. Nevertheless, similar exon–intron architecture was observed among the clades of collated *At*SCC and *Os*SCC biosynthetic genes. The number of EN in *At*SCC and *Os*SCC biosynthetic genes ranged from 1 to 13 (Fig. 4). The *At*BGLU34 (*At1g47600*), *Os*6BGLU24 (*LOC_ Os06g21570*) and *Os*BGLU27 (*LOC_Os08g39860*) biosynthetic genes showed the highest exon and intron distribution with EN = 13 and IN = 12, respectively. There were eight SCC-encoding biosynthetic genes with EN = 1 and EN = 3, followed by seven SCC-encoding biosynthetic genes with EN = 2, five SCC-encoding biosynthetic genes with EN = 4–7, and three SCC-encoding biosynthetic genes with EN = 11.

The exon–intron architecture of *At*SCC and *Os*SCC syntenic gene pairs are described as follows: the *At*NIT2-*Os*NIT2 syntenic gene pair in clade 1 shared a similar number of exons (EN = 5), whereas, in *Os*IPMDH-*At*IMD1/3 syntenic gene pair, a total of 11 exons were distributed in *Os*IPMDH and about 8–9 exons in *At*IMD1 and *At*IMD3. In sub-clade 4, the *Os*BGLU24 and *Os*BGLU27 biosynthetic genes displayed 13 exons as that of the *At*BGLU34 biosynthetic gene except for *Os*BGLU29, *Os*BGLU9, and *Os*BGLU35 (EN = 6–7). The EN in the remaining clades displayed a similar trend; *At*SOT18-*Os*SOT in clade 8 (EN = 1), *At*ACO4-*Os*FLS1/*Os*2ODD25 and *At*GSL-OH—*Os*2ODD25 in clade 9 (EN = 3) (Fig. 4). Four non-syntenic genes with the same exon number are present in clades 6 and 8. The following syntenic gene pairs displayed dissimilarities in the EN: (i) *At*ACO4-*Os*HIS1, (ii) *At*ACO9-*Os*2ODD16, (iii) *At*ACO8-*Os*HIS1, (iv) *At*ACO8-*Os*2ODD16, (v) *At*IGMT5-*Os*COMTL4 and (vi) *At*IGMT1-*Os*COMTL5. The rice *Os*HIS1, *Os*2ODD16, *Os*COMTL4 and *Os*COMTL5 biosynthetic genes gained one exon, while their syntenic pairs *At*ACO4, *At*ACO8, *At*ACO9, *At*IGMT1 and *At*IGMT5 lost one exon. Two exon gains were observed in *Os*IPMDH, *Os*IPMS1, and *Os*IPMS2 biosynthetic genes, in contrast to two exon losses in each *At*IMD1 and *At*MAM1 biosynthetic genes. A total of 25 rice SCC-encoding biosynthetic genes in established synteny and similarity against motifs distributions and exon–intron structure of 18 *At*SCC biosynthesis genes (Table 1).

**Table 1:** Mining for *Oryza sativa* sulfur-encoding biosynthetic genes (*OsSCC)* with Arabidopsis sulfur-encoding biosynthetic gene (*AtSCC)* input data. Selection criteria are described as following: (1) synteny events; (2) phylogenetic clade; (3) motif composition (Os/At); and (4) number of exon (EN) with *AtSCC* biosynthetic genes (Os/At).

Selection of *OsSCC* biosynthetic genes						
*AtSCC* ID	*OsSCC* ID	*OsSCC* name	Criteria			
			1	2	3	4
At3g44300 (*AtNIT2*)	LOC_Os02g42330	*OsNIT2*	7.00E-158	1	3/5	5/5
At1g31180 (*AtIMD3*)	LOC_Os03g45320	*OsIPMDH*	0.00E-000	1	10/10	11/8
At5g14200 (*AtIMD1*)	LOC_Os03g45320	*OsIPMDH*	0.00E + 00	1	10/10	11/9
At1g47600 (*AtBGLU34*)	LOC_Os09g31410	*OsBGLU29*	2.00E-149	4	7/9	6/13
	LOC_Os08g39860	*OsBGLU27*	3.00E-158	4	9/9	13/13
	LOC_Os06g21570	*OsBGLU24*	1.00E-156	4	9/9	13/13
	LOC_Os04g39814	*OsBGLU9*	1.00E-82	4	7/9	7/13
	LOC_Os11g08120	*OsBGLU35*	2.00E-41	4	5/9	7/13
At1g74090 (*AtSOT18*)	LOC_Os09g08190	*OsSOT*	8.00E-66	8	4/4	1/1
At1g03400 (*AtACO4*)	LOC_Os06g14390	*OsACO4*	1.00E-84	9	7/9	2/3
	LOC_Os02g17940	*OsHIS1*/*Os2ODD12*	1.00E-38	9	9/9	4/3
	LOC_Os03g63900	*Os2ODD26*	5.00E-37	9	9/9	1/3
At5g43440 (*AtACO9*)	LOC_Os06g14390	*OsACO4*	5.00E-75	9	7/9	2/3
	LOC_Os09g18450	*OsFLS1*	3.00E-111	9	9/9	3/3
	LOC_Os01g24980	*Os2ODD16*	7.00E-50	9	9/9	4/3
	LOC_Os03g63900	*Os2ODD26*	4.00E-46	9	9/9	1/3
	LOC_Os03g32470	*Os2ODD25*	1.00E-35	9	9/9	3/3
At5g23010 (*AtMAM1*)	LOC_Os11g04670	*OsIPMS1*	7.00E-173	9	1/1	12/10
	LOC_Os12g04440	*OsIPMS2*	5.00E-173	9	1/1	12/10
At3g61400 (*AtACO8*)	LOC_Os02g17940	*OsHIS1*/*Os2ODD12*	1.00E-34	9	9/9	4/3
	LOC_Os01g24980	*Os2ODD16*	3.00E-39	9	9/9	4/3
At2g25450 (*AtGSL-OH*)	LOC_Os03g32470	*Os2ODD25*	8.00E-23	9	9/9	3/3
At1g76790 (*AtIGMT5*)	LOC_Os02g57760	*OsCOMTL4*	1.00E-40	10	7/8	4/3
At1g21100 (*AtIGMT1*)	LOC_Os08g06100	*OsROMT9*	6.00E-92	10	8/8	2/3
	LOC_Os04g09604	*OsCOMTL5*	3.00E-74	10	8/8	4/3
At2g43100 (*AtIPMI2*)	LOC_Os02g43830	*OsSta2*	2.00E-067	11	1/1	1/1
At1g24100 (*AtUGT74B1*)	LOC_Os11g04860	*OsUGT75E1*	4.00E-058	11	7/8	1/2
	LOC_Os09g11290	*OsIAGLU*	2.00E-12	11	2/8	1/2
At2g22330 (*AtCYP79B3*)	LOC_Os04g08824	*OsCYP79A10*	N/A	6	9/10	3/3
At4g39950 (*AtCYP79B2*)	LOC_Os04g08824	*OsCYP79A10*	N/A	6	9/10	3/3
At5g61290 (*AtFMOGS-OX-like8*)	LOC_Os10g40570	*OsFMOGS-OX-like5*	N/A	8	8/8	7/7
At5g07800 (*AtFMOGS-OX-like9*)	LOC_Os10g40570	*OsFMOGS-OX-like5*	N/A	8	8/8	7/7
At1g24100 (*AtUGT74B1*)	LOC_Os06g23800	*OsFMOGS-OX*	N/A	11	1/1	6/2

### Gene ontology (GO) and KEGG pathway enrichment of SCC-encoding biosynthetic genes

The GO and pathway enrichment analysis of rice and *Arabidopsis* SCC-encoding biosynthetic genes revealed a total of 206, 149 and 37 hits (terms) in biological process (BP), molecular function (MF) and KEGG pathway, respectively. The number of hit terms, commonly enriched among the rice and *Arabidopsis* SCC-encoding biosynthetic genes are as follows: BP; 30, MF; 34 and KEGG pathway; 9. In BP, the most significantly enriched terms among the rice SCC-encoding biosynthetic genes are sulfation, hormone biosynthetic process and hormone metabolic process whereas, in *Arabidopsis* SCC biosynthetic genes, the following terms were significantly enriched: (i) S-glycoside metabolic process, glycosinolate metabolite process and glucosinolate metabolic processes. In both the rice and *Arabidopsis* SCC biosynthetic genes, sulfur compound metabolic process and organic acid metabolic process were commonly present.

In MF, oxidoreductase activity, acting on paired donors with incorporation or reduction of molecular oxygen was the most significantly enriched (with more than 180 hits) term in both rice and Arabidopsis SCC biosynthetic genes. Other terms enriched at a relatively high extent are as follow: (i) oxidoreductase activity, acting on paired donors, with incorporation or reduction of molecular oxygen, NAD(P)H as one donor and incorporation, (ii) monooxygenase activity, (iii) iron ion binding, (iv) heme binding, (v) tetrapyrrole binding, (vi) metal ion binding and (vii) N, N-dimethylalanine monooxygenase activity.

The KEGG pathway enrichment showed involvement of the rice-*Arabidopsis* homologous genes in 10 different signalling pathways. The highest number of genes were significantly enriched in the metabolic pathways and biosynthesis of secondary metabolites with a total number of genes of 80 and 67, respectively. The tryptophan metabolism and 2-oxocarboxylic acid metabolism were fairly high at 25 and 21, respectively (Fig. 6).

### Chromosomal distributions of the SCC biosynthetic genes in *A. thaliana* and *O. sativa*

**Figure 5:**
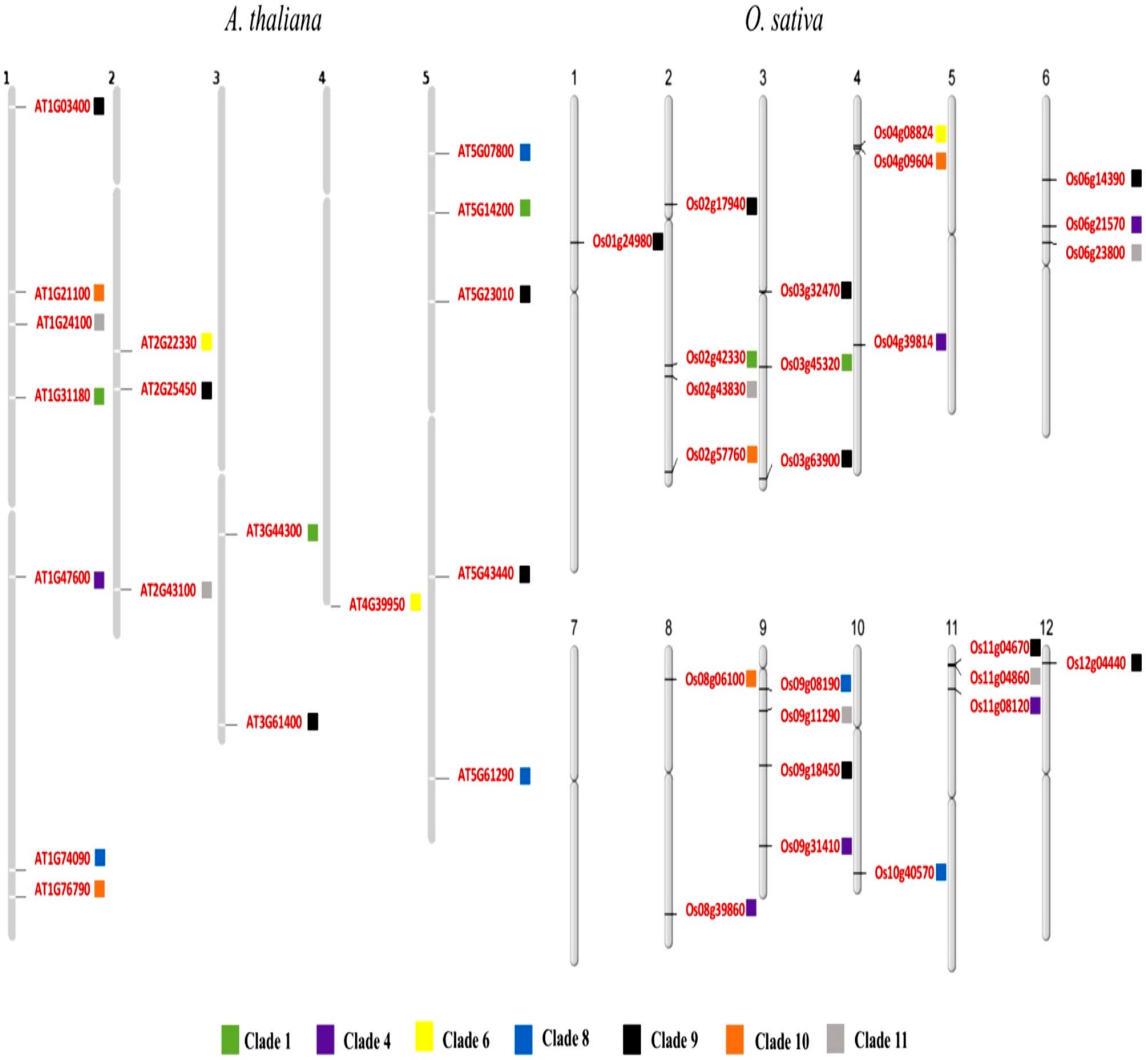
Sulfur-containing compound (SCC) encoding biosynthetic gene distribution in *A. thaliana* and *O. sativa* chromosomes. Grey bars represent the physical maps. The chromosomes are numbered accordingly: *A. thaliana*;1–5 and *O. sativa*;1–12. Short lines on grey bars represent the locations of SCCs biosynthetic genes (labelled in red) on each physical map. The different colour boxes expressed adjacent to the gene ID represent the clades.

Highly conserved SCC biosynthetic genes were physically mapped on the *Arabidopsis* and rice genomes. The SCC biosynthetic gene distribution in *Arabidopsis* and rice chromosomes are unequal (Fig. 5). In *Arabidopsis*, chromosome 1 showed the highest gene number (GN) = 10, followed by chromosome 5 (GN = 8), chromosome 2, (GN = 5), chromosome 3 (GN = 3) and chromosome 4 (GN = 1). The rice SCC biosynthetic genes are distributed in all the 12 chromosomes except chromosomes 5 and 7. Chromosomes 1, 3, 4, 6, 8, 10, 11 and 12 contain one to three *Os*SCC biosynthetic genes, and the highest number of *Os*SCC biosynthetic genes (GN = 4) are distributed on chromosomes 2 and 9. No tandem duplications are observed among the SCC biosynthetic genes; no two gene loci are arranged in close proximity and genes are separated by more than five duplicated blocks (Fig. 5). The *Os*IPMS1 and *Os*IPMS2 encoding proteins have the longest protein length (635 aa) in rice and *At*CYP79B3 (543 aa) in *Arabidopsis*. *Os*IAGLU and *At*IPMI2 are the shortest protein-encoding gene in rice (113 aa) and *Arabidopsis* (256 aa), respectively. More than half of the proteins encoded by the SCC biosynthetic genes are acidic, with a theoretical pI (isoelectric point) ranging from 4.63 to 6.24 (*Arabidopsis*) and 5.1 to 6.8 (rice). The average molecular weight (MW) of *At*SCC biosynthetic genes is 45.94 kDa and 43.24 kDa for the *Os*SCC biosynthetic genes (Table 2).

**Table 2:** Sulfur-encoding biosynthetic gene, chromosomal and protein level description in Arabidopsis and rice. Each gene is characterized according to its chromosome number, chromosomal loci, open reading frame (ORF) and physical characteristics of the encoding protein.

Gene ID	Gene name	Chr	Location	ORF length (bp)	Protein		
					Length	PI	MW (kDa)
AT1G03400	*AtACO4*	1	842,747–844,190	1056	351	6.15	39.13
AT1G21100	*AtIGMT1*	1	7,386,839–7,388,428	1122	373	5.01	40.869
AT1G24100	*AtUGT74B1*	1	8,525,435–8,527,087	1383	460	4.63	51.002
AT1G31180	*AtIMD3*	1	11,142,714–11,144,633	1215	404	5.55	43.847
AT1G47600	*AtBGLU34*	1	17,491,732–17,494,759	1536	511	8.21	57.542
AT1G74090	*AtSOT18*	1	27,862,909–27,864,193	1053	350	5.5	40.465
AT1G76790	*AtIGMT5*	1	28,822,186–28,823,673	1104	367	4.76	40.222
AT2G22330	*AtCYP79B3*	2	9,488,554–9,491,187	1632	543	8.17	61.437
AT2G25450	*AtGSL-OH*	2	10,829,916–10,831,655	1080	359	6.24	40.351
AT2G43100	*AtIPMI2*	2	17,920,660–17,921,689	771	256	6.01	27.043
AT3G44300	*AtNIT2*	3	15,983,311–15,985,535	1020	339	5.24	37.153
AT3G61400	*AtACO8*	3	22,718,956–22,720,397	1113	370	5.64	41.601
AT4G39950	*AtCYP79B2*	4	18,525,246–18,527,579	1626	541	8.73	61.347
AT5G07800	*AtFMOGS-OX-like9*	5	2,486,576–2,489,296	1383	460	6.21	52.337
AT5G14200	*AtIMD1*	5	4,576,202–4,578,402	1230	409	5.81	44.161
AT5G23010	*AtMAM1*	5	7,703,092–7,706,896	1521	506	7.28	55.125
AT5G43440	*AtACO9*	5	17,455,233–17,456,657	1098	365	6.18	40.86
AT5G61290	*AtFMOGS-OX-like8*	5	24,648,558–24,650,815	1386	461	4.9	52.406
LOC_Os01g24980	*Os2ODD16*	1	14,077,629–14,080,716	1035	344	5.62	38.731
LOC_Os10g40570	*OsFMOGS-OX-like5*	10	21,724,416–21,727,181	1449	482	5.69	53.726
LOC_Os11g04670	*OsIPMS1*	11	1,989,201–1,995,087	1908	635	6.46	68.448
LOC_Os11g04860	*OsUGT75E1*	11	2,067,727–2,069,430	1449	482	5.38	54.068
LOC_Os11g08120	*OsBGLU35*	11	4,262,908–4,265,304	579	197	9.81	22.062
LOC_Os12g04440	*OsIPMS2*	12	1,888,943–1,894,920	1908	635	6.46	68.461
LOC_Os02g17940	*OsHIS1/Os2ODD12*	2	10,386,279–10,390,290	1056	351	5.1	40.118
LOC_Os02g42330	*OsNIT2*	2	25,459,397–25,462,730	1074	357	5.75	37.985
LOC_Os02g43830	*OsSta2*	2	26,465,591–26,469,280	774	257	7.61	26.443
LOC_Os02g57760	*OsCOMTL4*	2	35,370,515–35,373,858	1098	365	5.34	38.647
LOC_Os03g32470	*Os2ODD25*	3	18,570,651–18,572,508	1650	549	8.36	60.587
LOC_Os03g45320	*OsIPMDH*	3	25,586,205–25,590,717	1227	408	5.86	43.371
LOC_Os03g63900	*Os2ODD26*	3	36,103,513–36,105,068	1089	362	5.97	40.792
LOC_Os04g08824	*OsCYP79A10*	4	4,869,932–4,872,151	1476	491	9.26	55.727
LOC_Os04g09604	*OsCOMTL5*	4	5,161,917–5,167,494	1137	378	5.33	40.594
LOC_Os04g39814	*OsBGLU9*	4	23,715,443–23,721,731	951	316	6.3	35.548
LOC_Os06g14390	*OsACO4*	6	8,031,719–8,035,243	1098	365	5.23	39.169
LOC_Os06g21570	*OsBGLU24*	6	12,437,997–12,442,742	1515	504	7.18	57.756
LOC_Os06g23800	*OsFMOGS-OX*	6	13,905,082–13,909,018	711	236	8.94	25.725
LOC_Os08g06100	*OsROMT9*	8	3,337,751–3,340,959	1107	368	5.41	39.75
LOC_Os08g39860	*OsBGLU27*	8	25,250,314–25,254,656	1500	499	8.53	56.804
LOC_Os09g08190	*OsSOT*	9	4,250,758–4,251,917	843	280	6.8	31.922
LOC_Os09g11290	*OsIAGLU*	9	6,266,198–6,266,539	342	113	5.25	12.531
LOC_Os09g18450	*OsFLS1*	9	11,309,063–11,310,776	1050	349	6.19	39.001
LOC_Os09g31410	*OsBGLU29*	9	18,889,721–18,893,801	1401	466	9.02	53.08

## Discussion

Sulfur (S) is a secondary macronutrient that regulates plant physiology, growth and developmental processes such as photosynthesis, biosynthesis of sulfur-containing compounds (SCCs) and hormone biosynthesis. It is the 4th major nutrient for crop production after nitrogen, phosphorus and potassium. In higher plants, the S acquisition and assimilation consumes high energy. The S element is taken up by plants as sulphate ions mainly via roots and a small amount can be absorbed through leaves. In rice, the S element, S-containing genes and associated SCCs are critically involved in stress-responsive mechanisms^45^.

For example, the glutathione S-transferase (GST), a detoxification enzyme ubiquitously present in vertebrates and invertebrates plays an important role in xenobiotic compound detoxification. GST activity is associated with oxidative stress protection as it acts as a mediating substrate in various biochemical reactions, interacts with phytohormones and redox metabolites, and coordinates stress-induced signalling events^10^. Glutathione (GSH) mediates abiotic and biotic stress resistance using the ROS-scavenging mechanism of the first defense line system in crop plants^46^. Extensive studies have evident GSH-mediated tolerance mechanisms against salinity, drought, heavy metal toxicity, chilling and herbicides in rice, wheat, barley, soybean and canola^47^. The effect of S amendment on plant defense response had contributed to similar evidence. As such, the soil amendment of S-containing fertilizer on wheat varieties increased resistance against brown rust and improved the overall productivity^48^.

Rice yield-impeding factors include pest and pathogen, climate, weather, soil infertility, heavy metal contamination and others. Presently, rice yield enhancement strategies are vigorously carried out by tapping into various aspects of rice biology. Genetic studies, molecular breeding, genetic engineering, heterosis breeding and population improvement are amongst the most sought-after tools utilized in modern rice breeding^49–51^. Since a large number of studies on rice S and SCCs have been linked to stress mechanisms and defense responses, a comprehensive annotation of SCC-encoding genes in the rice genome is important to necessitate enhanced manipulation strategies in breeding approaches^52–56^.

In this study, a total of 665 *Os*SCC biosynthetic genes were identified as the homologs of *At*SCC query sequences. A total of 477 syntenic gene pairs (*Arabidopsis*-rice) and 25 rice SCC biosynthetic genes (*At*SCC homologs) were obtained using a comprehensive analysis entailing synteny, phylogenetic, conserved motif distribution and gene structure. The synteny analysis identified the gene order and compared the genomic structural changes of the target genes. Shared synteny assumes a common ancestor/evolutionary origin and a syntenic fragment shares a similar function^57,58^. A small number of genes identified as *Arabidopsis*-rice syntenies , suggests the early Angiosperm divergence of monophyletic monocot from its eudicot relatives^59^. The monocot rice genome with 5 chromosomes typically diverged from the eudicot *Arabidopsis* genome (7 chromosomes) of a higher chromosome number^60^. The synteny analysis of *Arabidopsis*-rice SCC biosynthetic genes implies the ancient existence of SCC biosynthetic genes, even before the divergence of the *Arabidopsis*-rice (eudicot-monocot).

The SCC biosynthetic gene distribution pattern suggests the occurrence of an expansion event during evolution which could have possibly gone through gene co-localization or inter-chromosomal translocation^61^. The phylogenetic and gene structure pattern of the SCC-encoding biosynthetic genes suggest exon loss and gain events during *Arabidopsis*-rice (eudicot-monocot) evolution. The exon–intron arrangement pattern in 25 *At*SCC and 18 *Os*SCC suggests that the species-specific genome features are conserved^62^. The mosaic patterning of the SCC gene exon–intron regions could be associated with evolutionary forces that shaped the SCC biosynthetic gene structure dynamics.

Motifs are frequently occurring (conserved) regions within a DNA sequence. Found within the regulatory regions such as promoters and 3î UTRs, the 4–10 base pair motifs carry significant genome regulatory functions. Two species are likely to be close relatives if they share a high content of common motifs^63^. During speciation, mutations lead to either an accumulation or loss of motifs (motif turnover) and thus, a motif content analysis is often regarded as more advantageous than the counterpart sequence similarity search analysis. Our results showed that at least 10 different motifs identified in the Arabidopsis and rice SCC-encoding biosynthetic genes have similar distribution patterns by clades.

For instance, in clade 1, six motifs were annotated as 3-isopropylmalate dehydrogenase despite differences in the DNA and protein sequences. Likewise in Clade 4, about 7 different motifs are annotated as glycosyl hydrolase family 1 whereas, in Clade 10, there are 4 motifs corresponding to O-methyltransferase domain (Supplementary 2). The *Os*SCC biosynthetic genes identified in this study showed potential functional roles in plant defense response. In clade 1, *LOC_Os02g42330* (nitrilase 1), the syntenic pair of *At3g44300* (nitrilase 2) was reported to participate in the tryptophan-dependent pathway of auxin biosynthesis in rice^64^. Three *Os*SCC biosynthetic genes from clade 10 were characterized as O-methyltransferase, a key gene in *Arabidopsis* indolic glucosinolate modification. As shown in Table 1, five -glucosidase genes from clade 4 showed syntenies with glucosidase 34 (AtBGLU34). AtBGLU34 plays a major role in response to salt stress^65^ and indolic glucosinolate biosynthesis^66^ in *Arabidopsis*.

**Figure 6:**
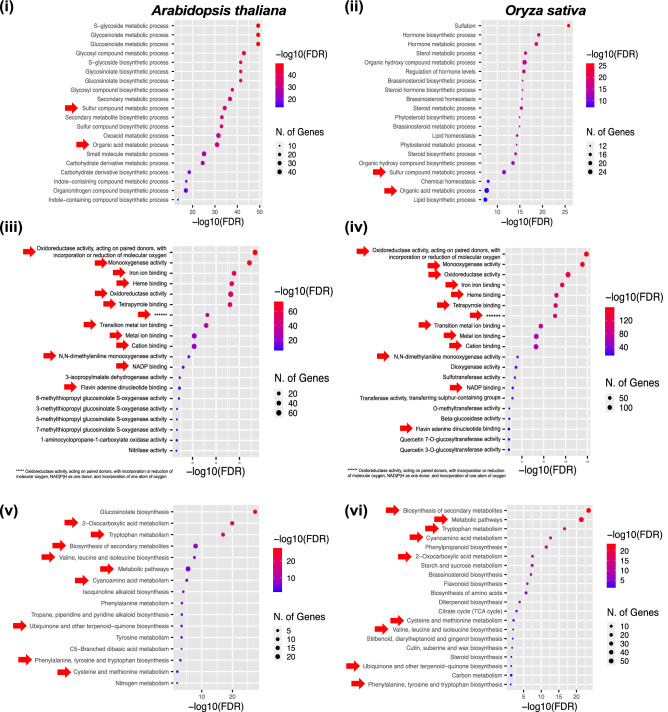
Gene ontology (GO) and pathway enrichment analysis. The bubble plot represents the top 20 significantly enriched terms of the *Arabidopsis-*rice homologous SCC-encoding geens. The GO terms are presented in (i-ii) biological process and (iii-iv) molecular functions whereas the KEGG pathways are presented in (v-vii). Red arrows represent the terms shared among the *Arabidopsis-*rice orthologous genes. The results are visualized at P < 0.05 using ShinyGO v0.75 (http://bioinformatics.sdstate.edu/go75/).

The SCC biosynthetic genes distributed among the unique phylogenetic clades, carrying similar motif pattern are possibly sharing a similar function. The unique motifs in each clade could be associated with specific functional roles of the SCC biosynthetic genes. The current findings shed insights on the potential functional roles of SCC biosynthetic genes in rice as more than half of the genes were putatively involved in the biosynthesis of aliphatic glucosinolate and indolic glucosinolate. Based on the gene ontology and pathway enrichment analysis, the *Arabidopsis*-rice homologous SCC-encoding genes were significantly enriched in the sulfur compound metabolic process (BP), oxidoreductase activity, acting on paired donors with incorporation or reduction of molecular oxygen (MF) and biosynthesis of secondary metabolites (KEGG pathway) (Fig. 6). This may suggest the role of the SCC-encoding genes in S assimilation, whereby the reduction of sulphate ion to sulphide and subsequent S-containing amino acids (methionine and cysteine) via the adenosine phosphosulphate pyrophosphate (APS) and phosphoadenosine phosphosulphate (PAPS) is catalyzed by the participating enzyme activities.

In plant breeding strategies, exploiting the naturally occurring genetic variation is of utmost fundamental in controlling genes of agronomic importance. Physical maps of rice SCC biosynthetic genes provided in this study could be harnessed for chromosomal region manipulated breeding techniques such as the target chromosome-segment substitution^67^ and hotspot chromosomal regional positioning of desirable candidate genes^68^. The findings enable the selection of desirable target rice genes which are tightly linked to S and SCC-encoding genes with a putative functional role in stress response mechanisms.

## Conclusions

Rice SCCs biosynthetic genes show syntenic associations with *Arabidopsis* homologs (*At*SCCs). The high degree of conservation between the *At*SCC and *Os*SCC genes suggests long conservation history which could be implicated in SCC gene functions in plant defense response. The present findings not only identified the rice SCC-encoding genes (*Os*SCC) but also stretch further to include chromosomal level-mapping to better inform new directions in rice functional research and breeding manipulation strategies.

## Supplementary Information

Supplementary Information 1.

Supplementary Information 2.

## Data Availability

All open-source genomic datasets analysed in this study are available in the Phytozome v13.0 database (https://phytozome-next.jgi.doe.gov/), *Arabidopsis* Information Resource v10.0 (TAIR) (https://www.arabidopsis.org) and *O. sativa* Genome Annotation Project Database v7.0 (RGAP) (http://rice.uga.edu/).
